# The Porcine TSPY Gene Is Tricopy but Not a Copy Number Variant

**DOI:** 10.1371/journal.pone.0131745

**Published:** 2015-07-02

**Authors:** Anh T. Quach, Olutobi Oluwole, William Allan King, Tamas Revay

**Affiliations:** Department of Biomedical Sciences, Ontario Veterinary College, University of Guelph, Guelph, Ontario, Canada; University of Bologna, ITALY

## Abstract

The testis-specific protein Y-encoded (TSPY) gene is situated on the mammalian Y-chromosome and exhibits some remarkable biological characteristics. It has the highest known copy number (CN) of all protein coding genes in the human and bovine genomes (up to 74 and 200, respectively) and also shows high individual variability. Although the biological function of TSPY has not yet been elucidated, its specific expression in the testis and several identified binding domains within the protein suggests roles in male reproduction. Here we describe the porcine TSPY, as a multicopy gene with three copies located on the short arm of the Y-chromosome with no variation at three exon loci among 20 animals of normal reproductive health from four breeds of domestic pigs (Piétrain, Landrace, Duroc and Yorkshire). To further investigate the speculation that porcine TSPY is not a copy number variant, we have included five Low-fertility boars and five boars with exceptional High-fertility records. Interestingly, there was no difference between the High- and Low-fertile groups, but we detected slightly lower TSPY CN at all three exons (2.56-2.85) in both groups, as compared to normal animals, which could be attributed to technical variability or somatic mosaicism. The results are based on both relative quantitative real-time PCR (qPCR) and droplet digital PCR (ddPCR). Chromosomal localization of the porcine TSPY was done using fluorescence in situ hybridization (FISH) with gene specific PCR probes.

## Introduction

The majority of the Y-chromosome represents a special part of the mammalian genome, that is unique to males. A multitude of evolutionary rearrangements, deletions, inversions, transpositions and amplifications, are thought to have eroded its size and shaped its structure [1]. It is not surprising that this resulted in highly variable Y-chromosomal structures, as showed by the comparison of few completely sequenced, assembled and mapped examples of Y-chromosomes which include human, chimpanzee, rhesus monkey and mouse [2–5]. On the other hand, it also contains the SRY gene, the essential sex determination locus, as well as several genes expressed specifically in the testis and potentially with substantial functions in gametogenesis and fertility [1,2].

The testis-specific protein Y-encoded (TSPY) gene is situated on the male specific portion of the mammalian Y-chromosome and exhibits some remarkable biological characteristics. Foremost, the TSPY has the highest copy number (CN) of all protein coding genes in the human genome (~35, [6]). This exceptional CN is surpassed in domestic cattle bulls which have on average 94 copies of TSPY [7]. While most mammalian genes exist and function in two copies, recent discovery of copy number variations (CNV) identified that those genes could be affected by deletions or duplications resulting in ± 1–2 copies [8]. This is not the simple case for TSPY, as the variation among men and bulls ranges between 11–76 [9], and 50 to 200, respectively [7]. This variation could be explained by the tandem repeated array structure they lie within the Y-chromosome, that could provide opportunities for imperfect pairing and recombination between the sister chromatids, resulting in large deletions or additional copies [10]. This tandem repeated genomic structure—named simply as the TSPY cluster (although individual copies have an additional numerical identifier) represents the largest and most homogenous protein coding cluster in the human genome [2].

TSPY orthologs were identified in many other species with strikingly different copy numbers from the human and bovine examples. The chimpanzee and rhesus monkey Y-chromosomes contain six copies of TSPY, while multi copy status has also been suggested in other great apes, horses, cats and dogs [4,5,11,12]. Interestingly rats have only one functional copy, while mice have lost the functional TSPY locus [13,14]. The structure of the porcine TSPY locus is not known, however a PCR identified BAC clone has been used as FISH probe and mapped to a single Y-chromosomal locus [15] but its copy number was not yet determined. There is only one predicted TSPY gene sequence identified in the various gene banks, interestingly named as TSPY4. Although the current Sscrofa10.2 genome does not contain a Y-chromosome assembly, this TSPY4 mRNA aligns perfectly to the whole Y-chromosomal draft sequence.

Neither the biological function of TSPY, nor the effect of its variable copy number has been clearly elucidated. The conservation of the protein sequence across many mammals and its specific expression in the testis suggests roles in male reproduction. Molecular analysis identified several binding domains to interact with cyclins and EEF1A, so functions in regulation of cell cycle, renewal of spermatogonia and also as proto-oncogene in various testicular cancers have been proposed [16,17]. It is also mapped at the putative gonadoblastoma locus [18]. The impact of variation in TSPY copy number is not known, however association with fertility has been identified in cattle and humans [9,19,20].

There are TSPY paralogs identified outside the Y-chromosomal TSPY locus. The human genome contains six TSPY-like genes, the five autosomal copies are most probably derived by retrotransposition, while the one copy on the X-chromosome marks the common evolutionary origin of the sex chromosome pair [9]. Interestingly studies suggest contrasting functions of TSPX as tumor supressor compared to TSPY, based on its expression pattern and molecular interactions [9]. The role of the autosomal TSPY-like genes is not clear, however a mutation of TSPY-L1 identified in a case of sudden infant death syndrome, suggests involvement in testis differentiation [21]. There are two autosomal (TSPYL1, TSPYL4) and one X-chromosomal (TSPYL2) paralogs identified in the current porcine genome, but their functions are unknown.

The purpose of this study was to characterize the porcine Y-chromosomal TSPY gene through the detection of several loci along the gene, determine its copy number and CNV status. Here we report, for the first time the copy number of TSPY in four breeds of domestic pig.

## Materials and Methods

### Animals and DNA extraction

Peripheral blood samples from 20 unrelated boars of normal reproductive health from four breeds (5 from each of Piétrain, Landrace, Duroc and Yorkshire) and a female control animal were obtained from local producers. Sampling was done as part of the general animal health check and mandatory sampling for CFIA (Canadian Food Inspection Agency) tests, according to the Canadian Council on Animal Care and University of Guelph’s Animal Care Committee guidelines by licensed veterinarians. These animals were not selected for research purposes, but regular breeding animals at various Canadian farms and leftover blood samples were used for DNA extraction using standard phenol-chloroform technique [22] and concentrations were determined by a NanoDrop Spectrophotometer.

In order to extend the panel of the 21 tested animals, of which we had no specific fertility information available other than their general good reproductive health, 10 additional unrelated boars with known fertility status (five High-fertility and five Low-fertility) were chosen from a separate population [23]. The fertility indicator parameter was the Direct Boar Effect on litter size (DBE) that is the number of piglets the given boar produces in average per litter (S3 Table) as compared to the overall average of the population and corrected for all identified environmental effects and breeding values of their mate [24]. DNA samples of these animals were retrieved from the owner’s DNA bank. It should be noted that the method of DNA extraction and the age of the DNA samples were variable and could not be controlled by this study.

### Relative TSPY copy number determination by qRT-PCR

**Fig 1:**
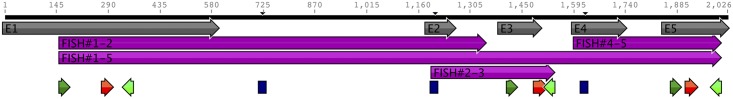
Schematic view of the porcine TSPY gene (exons 1–5 in grey) and location of primers (green), TaqMan probes (red), PflMI enzyme cut positions (blue) and FISH probes (purple).

Quantitative real-time PCR (qPCR) was used to determine TSPY copy number (CN) relative to the androgen receptor gene (AR, NM_214314.2, localized on the X-chromosome: NC_010461.4), a single copy reference gene. The porcine TSPY gene sequence was identified using the Sscrofa10.2 genome assembly, that contains the raw Y-chromosome sequence (NC_010462) and is built from seven scaffolds. One of the scaffold (NW_003536871.2) contains the predicted TSPY4 gene (testis-specific Y-encoded 4-like, Gene ID: 100625034). The predicted mRNA sequence (XM_003360532.2) was aligned to the Y-chromosome DNA sequence and used as source for PCR assay development. Primers were designed to amplify exons 1, 3 and 5 of the TSPY gene (Fig 1) and exon 2 of AR using the Primer3 plug-in of Geneious software. Primer sequences, annealing conditions and product sizes are in S1 Table. qPCR was performed using a CFX96 Touch Real-Time PCR Detection System (Bio-Rad) under the following thermal profile: 98°C, 2 min; 49×(98°C, 15 sec; 60°C, 15 sec). Melting curve was then generated between 72°C to 95°C in 0.5°C/sec increments. The 10μl reaction mix consisted of 1× SsoFast EvaGreen Supermix (Bio-Rad), 2.25mM primers and 5ng genomic DNA. Samples were run in triplicate, an inter-run calibrator sample was selected (one Duroc boar) and included in all runs to minimize technical variations. The TSPY CN was determined relative to the calibrator sample (Eq 1) after normalization against the single copy reference gene using the efficiency corrected ΔΔCq formula (Eqs 2–3), as described in detail previously [7].

$$CN_{CAL}=\frac{E_{AR}^{Cq}}{E_{TSPY}^{Cq}}$$ (1)

$$Ratio=\frac{E_{TSPY}^{\Delta Cq}}{E_{AR}^{\Delta Cq}}$$ (2)

$$CN_{Sample}^{qPCR}=Ratio\times CN_{CAL}$$ (3)

where ΔCq = (Cq_CAL_-Cq_Sample_) is the difference of the threshold cycle values for Calibrator and the given Sample, while E is the primer efficiency for each amplicon. The efficiency values were calculated by two different ways. Method STD: from STD curves created from 2-fold serial dilutions (16ng—0.25ng) of DNA mixed from the 20 samples and analyzed in CFX manager (Bio-Rad). Method LinReg: the average of individual well efficiencies were calculated by linear regression of amplification curves using the LinRegPCR software [25,26].

### Absolute copy number determination by droplet digital PCR

Droplet digital PCR (ddPCR) was performed using the QX100 Droplet Digital PCR System (Bio-Rad). The TaqMan chemistry (5`FAM—internal ZEN—3`IowaBlack FQ quenchers) was used to detect the amplicons instead of the EvaGreen dye, used in qPCR above. TaqMan probes were designed to target TSPY exon 1, 3 and 5, as well as AR (Fig 1) using the PrimerQuest software (IDT). Prior to ddPCR 5μg DNA from each of 21 animals (the same 20 boars + one female control used for qPCR) were digested with FastDigest PfIMI restriction endonuclease (Thermo Scientific) in 50μl 1× FastDigest buffer for 5 min at 37°C, followed by 10 min inactivation at 65°C. This enzyme was chosen, as the simulated restriction digestion with all commercially available restriction endonuclease in Geneious software showed cut positions surrounding the PCR amplicons (TSPY, AR), but not within any of them (Fig 1). This digestion step helps to avoid tandem repeats to be encapsulated into the same droplet, thus causing false CN measurement. The ddPCR (Bio-Rad) is a three-step procedure [27]. First, the reaction mix (50ng digested DNA, 900nM primers, 250nM TaqMan probe, 1×ddPCR supermix in 25μl) is partitioned into 20,000 uniform 1-nl volume droplets using the QX100 droplet generator by mixing 20μl reaction mix and 70μl droplet oil (Bio-Rad) in the generator cartridge. Then 40μl of the resulting emulsion was transferred to a PCR plate and DNA was amplified under the following thermal cycle conditions: 94°C, 10 min; 45×(94°C, 30 sec; 60°C, 1 min with 2.5°C/sec ramp); 98°C, 10 min. The third step, the automatic signal detection was performed using the robotic droplet reader (Bio-Rad) that serially aspirate each sample, separates the droplets and detects the fluorescence in each droplet. Each sample was run in duplicates. The TSPY CN was calculated by dividing the measured target concentration (copies/μl,) with the concentration of the AR single copy reference gene (Eq 4, Quantasoft detection software, Bio-Rad).

$$CN_{Sample}^{ddPCR}=\frac{concentration_{TSPY}}{concentration_{AR}}$$ (4)

### Statistical analysis

For statistical analysis all data was subjected to D’Agostino-Pearson normality test, then comparisons of different groups were done using the Kruskal-Wallis test or two-way ANOVA. A p-value of less than 0.05 is considered significant. The data was analyzed using GraphPad Prism6 software (GraphPad Software).

### Fluorescence in situ hybridization (FISH)

Metaphase chromosome spreads were prepared from short term lymphocyte culture according to standard cytogenetic techniques. Whole blood (1.2ml) from the same boar used as calibrator for PCR was cultivated in 10ml RPMI 1640 (Invitrogen) medium containing 10% FBS (Invitrogen), 0.15% PenStrep (Invitrogen), 0.06% Phytohemagglutinin (Invitrogen) for 3 days at 37°C. KaryoMax Colcemid (0.025μg/ml, Invitrogen) was added to the culture for the last 25 minutes. Slides were prepared from the hypotonized (0.075M KCl, 20min, 37°C) and three times fixed (MeOH/AcOH, 3:1) suspensions and aged at room temperature for 3 days before using for FISH.

Four different biotinilated hybridization probes were prepared by PCR. The primers used for the copy number determination were combined across exons to result in different sized probes (Fig 1). Probes were named according to the exons content, as FISH#1–5 covered the whole length (1846bp), while FISH#1–2 (1187bp), FISH#2–3 (337bp) and FISH#4–5 (404bp), only a part of the gene. PCR mixes (50μl) contained 80 ng male genomic DNA, 5 Units AmpliTaq Gold DNA Polymerase (Applied Biosystems), 1×PCR Buffer II, 1.5 mM MgCl_2_, 200 μM each dATP, dCTP, dGTP, 133 μM dTTP, 67 μM biotin-11-dUTP (Metkinen) and 0.5 μM of each primer. All PCR reactions were performed in a MJ Research PTC-200 Thermo Cycler under the following thermal profile: 94°C, 10 min; 35×(94°C, 30 sec; 60°C, 30 sec; 72°C, 30 sec), 72°C, 10 min. PCR products were purified and concentrated to 20μl final volume using a Amicon Ultra 30K centrifugal filters (Millipore) before adding to the hybridization mixture, that was composed of 50% (v/v) formamide (Fisher), 10% (w/v) dextran-sulfate (Sigma-Aldrich), 15μg Salmon Sperm DNA (Life Technologies) and 2×SSC.

The FISH experiments were performed with standard protocols [28], briefly: slides were treated with pepsin, dehydrated in ethanol series, denatured at 72°C for 2 minutes in 70% formamide (Fisher)/2×SSC, then quenched in ice-cold ethanol. The hybridization mix was denatured at 72°C for 10 min before applying to the slide. After overnight hybridization at 37°C, slides were washed twice in Formamide/2×SSC (50:50), then twice in 0.2×SSC at 47°C and PBS/0.5% Tween 20 at room temperature for 3 minutes each. Slides were blocked in 1% BSA in PBS for 30 minutes at 37°C before detection. Biotinylated probe signals were visualized by one of the following two methods: 1. alternating layers of FITC-avidin (Vector, 1:400 in PBS/0.5% Blocking Reagent (Roche)) and anti-avidin-FITC antibody (Cambio, 1:250 in PBS/0.5% Blocking Reagent) incubated for 30 minutes at 37°C and washed by 3×(PBS/0.5% Tween 20) at room temperature. 2. Alternatively, the Tyramide signal amplification method was applied using Sterptavidin-HRP and AlexaFluor 594 tyramide (LifeTechnologies) according to the manufacturer’s protocol. Chromosomes were counterstained with DAPI (Sigma-Aldrich) and slides were mounted with Vectashield (Vector). Images were captured using a Leica DM5500B fluorescence microscope (Leica), equipped with a Retiga Exi Fast (QImaging) cooled digital camera and the OpenLab imaging software (Perkin Elmer).

## Results

### TSPY copy number by qPCR

We have measured the TSPY CN at three loci along the gene relative to AR by designing primers for exons 1, 3 and 5 (Fig 1). PCR amplification efficiencies were calculated for all these loci by two different methods (STD curve and LinRegPCR). PCR efficiencies of the TSPY-E1 assay (TSPY-E1 STD E = 1.89 and TSPY-E1 LinReg E = 1.82) were found to be different (Kruskal-Wallis test, p<0.01) from the other amplicons (AR, TSPY-E3, TSPY-E5), which were all very close to the theoretical ideal value of 2.00 (S1 Table).

**Fig 2:**
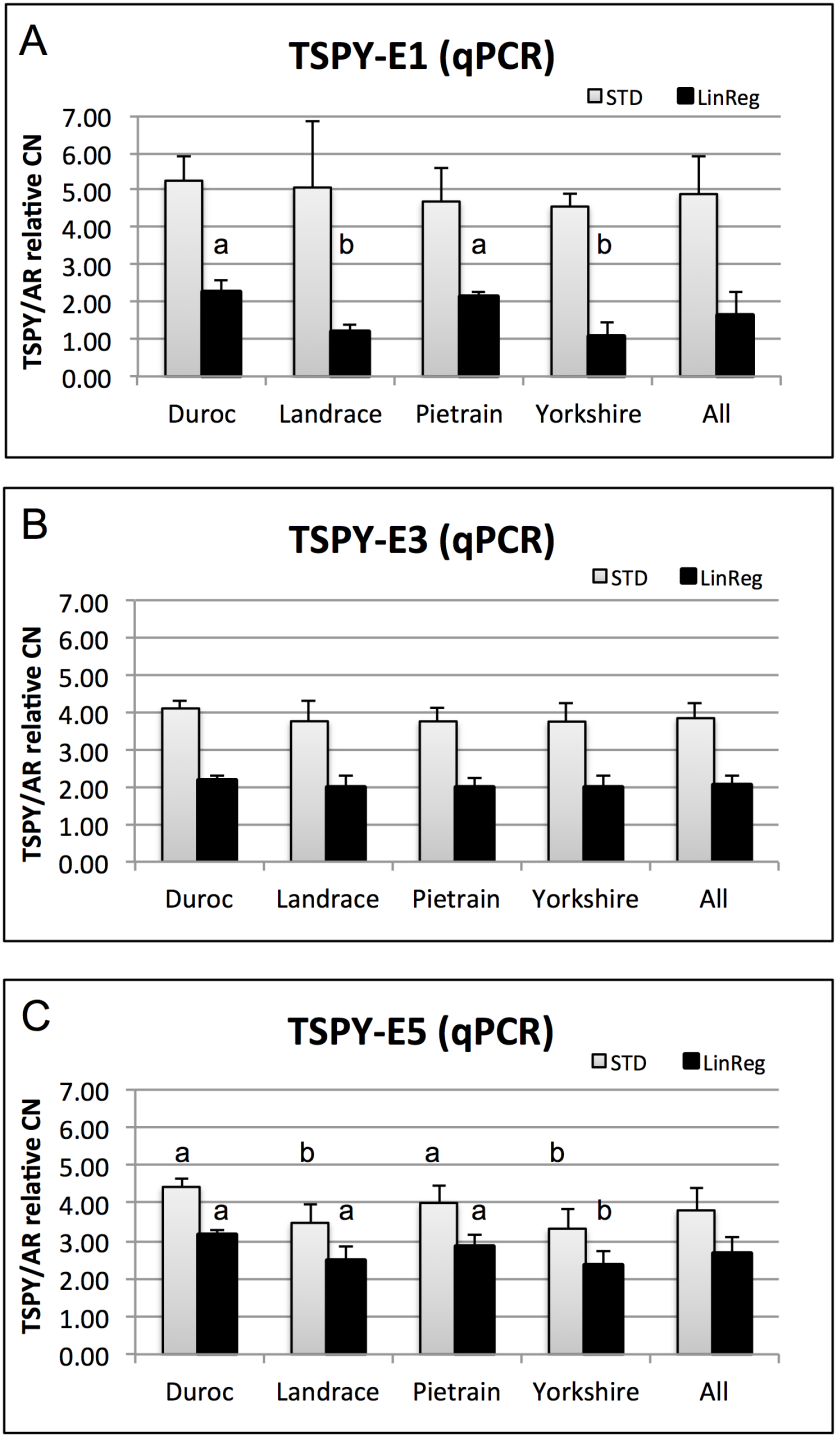
TSPY CN relative to the single copy AR gene, as determined by qPCR. CN was measured at three loci (Exon 1, 3, 5) in four breeds. Columns represent the average value (±SD) of 5 animals from the same breed. Different letters within efficiency calculation methods (STD or LinRegPCR) represent statistical significance according to two-way ANOVA (p<0.05).

The relative qPCR results in general, showed a highly variable TSPY CN ranging from 2–5 copies depending on the locus (E1, 3 or 5) or the method used for the calculation of PCR efficiency (STD or LinReg, Fig 2, S2 Table).

Significantly higher average copy numbers were calculated at all three exons (TSPY-E1 STD CN = 4.89±1.01, TSPY-E3 STD CN = 3.85±0.42, TSPY-E5 STD CN = 3.80±0.61) when the STD curves were used to determine PCR efficiency values, as compared to the TSPY copy number at the same locus calculated from LinRegPCR efficiencies (TSPY-E1 LinReg CN = 1.66±0.60, TSPY-E3 LinReg CN = 2.06±0.23, TSPY-E5 LinReg CN = 2.69±0.43).

The next level of comparison was among exons within either the STD or LinRegPCR efficiency calculation method. TSPY-E1 STD CN was significantly higher (4.89±1.01), than TSPY CN at exon 3 or 5 (TSPY-E3 STD CN = 3.85±0.42, TSPY-E5 STD CN = 3.80±0.61). However, TSPY-E5 was significantly higher (2.69±0.43) than the copy number at exons 1 and 3 (TSPY-E1 LinReg CN = 1.66±0.60, TSPY-E3 LinReg CN = 2.06±0.23) when the LinReg PCR was used to calculate efficiencies. This also suggests the dependency of CN calculation on efficiency values.

We have included Duroc, Landrace, Pietrain, Yorkshire boars to investigate potential breed specific CN variability. We found that the breeds showed very similar copy numbers at the same exon locus within STD or LinRegPCR calculations, with only a few exceptions, as labeled in Fig 2A and 2C.

### TSPY copy number by ddPCR

**Fig 3:**
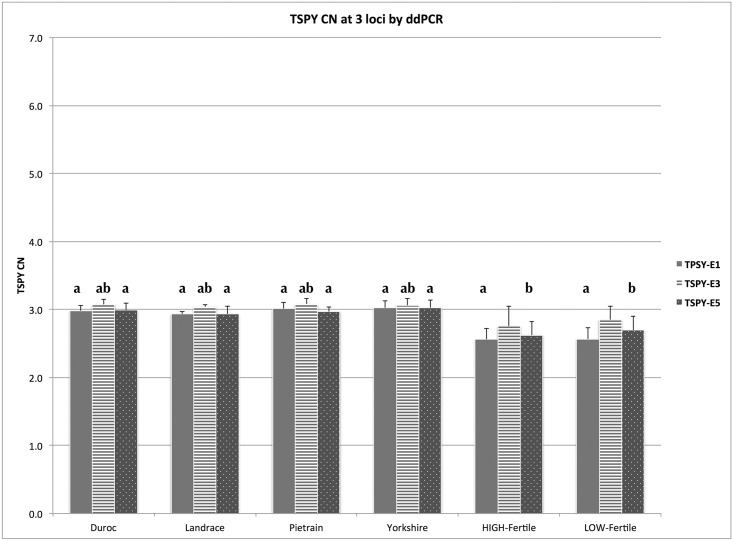
TSPY CN by ddPCR at exons 1, 3 and 5 in normal animals from four breeds, High-fertile and Low-fertile animals. Columns represent the average value (±SD) of 5 animals. Letters mark values that are statistically different (p<0.05).

The ddPCR experiments determined the porcine TSPY as a tricopy gene. The average TSPY CN among all boars tested was 3.01±0.08 and there were no differences among the three exons measured (TSPY-E1 CN = 2.99±0.08, TSPY-E3 CN = 3.06±0.07, TSPY-E5 CN = 2.98±0.10, Fig 3, S3 Table). The 20 boars, regardless of breed, showed no differences in TSPY CN (Fig 3).

We detected no positive droplets in the control female samples for all three exon specific TSPY primers, that clearly confirmed their specificity for only male specific Y-chromosomal sequences.

We found no CN difference among the High-fertile and Low-fertile animals at any of the three exons, however the values were generally slightly lower than that of normal animals, as measured by ddPCR (Fig 3, S3 Table). The average CN values for the High-fertile group were TSPY-E1: 2.56±0.16, TSPY-E3: 2.76±0.29, TSPY-E5: 2.62±0.2; similarly to the Low-fertile group averages at TSPY-E1: 2.57±0.16, TSPY-E3: 2.85±0.19, TSPY-E5: 2.70±0.21. The average CN at TSPY-E1 for both High- and Low-fertile groups were statistically lower than the average CN of normal animals at all three loci (Fig 3). The CN of TSPY-E5 in both High- and Low-fertile groups were also significantly lower than TSPY-E3 CN in normal animals.

### Chromosomal localization of TSPY by FISH

**Fig 4:**
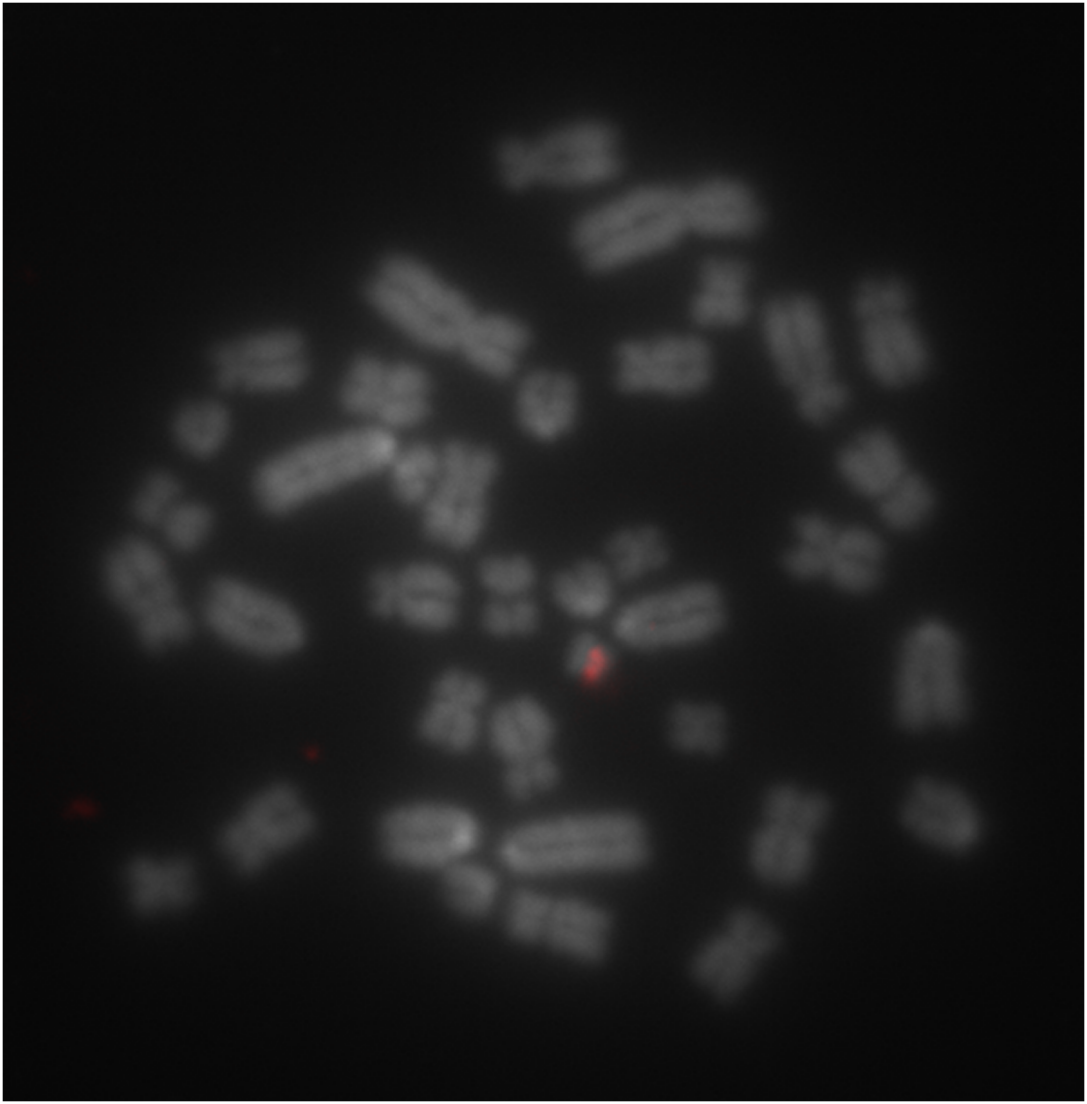
Y-chromosome specific fluorescence in situ hybridization signal of FISH probe#1–5 with Tyramide signal detection on a boar metaphase. DAPI stained metaphase chromosomes are converted grayscale and AF594 hybridization signal on the short arm of the Y-chromosome is red.

We performed FISH using 4 different sized probes (Fig 1). The two longest probes (FISH#1–5 and FISH#1–2) resulted in clear, intense and specific signals on the short arm of the Y-chromosome when the Tyramide signal detection method was applied (Fig 4). The same probes with the conventional avidin-FITC detection method and the two shorter probes (FISH#2–3, FISH#4–5) did not produce reliably detectable signals.

## Discussion

The structural arrangement of the porcine Y-chromosome is not fully characterized. The Sscrofa 10.2 genome assembly [29] contains the Y-chromosome sequence that is built from seven partially annotated scaffolds. The aim of our study was to determine the previously unreported Y-chromosomal TSPY gene copy number in pigs and investigate whether it is a copy number variant (CNV) among breeds and individual animals.

To ensure the male specificity of the GenBank predicted TSPY sequence that served as template for assay designs, we localized it by FISH on metaphase chromosomes. We chose to generate probes from combinations of the actual qPCR primers in order to avoid potential inclusion and detection of repetitive DNA from large-insert clones (Fig 1). Two of the four different probes produced signals specific for the short arm of the Y-chromosome (Yp, Fig 4), but an enhanced signal amplification method was needed [30]. This is still a surprising result, especially in light of the low TSPY copy number, as 1–2 kb long probes are generally suitable for physical mapping of highly repetitive sequences, in order to produce signals of detectable strength [28]. The specific localization of TSPY on Yp is in agreement with a previous study by Quilter et al. [15], who mapped TSPY among other genes by radiation hybrid panels and BAC clone based FISH.

The qPCR assays were designed to amplify three different exons (E1, 3, 5) of all the current TSPY sequences assigned to the porcine Y-chromosome. The investigation of three different loci along the TSPY allowed the detection of the potential partial amplification/deletion of the gene. This process occurs frequently on various loci of the Y-chromosome e.g. AZF regions, due to intrachromosomal recombination of repeated elements [31]. Primers were also tested and proved to be negative against female DNA to confirm that only Y-chromosomal TSPY gene copies and not autosomal or X-linked paralogs were detected.

We characterized the TSPY CN by qPCR, which is a frequently used technique for accurate copy number profiling, especially if the calculations are relative to a standard sample with known target CN [32]. To apply qPCR for CN discovery, thus without having any prior information on the TSPY CN, we used the methodology described by Hamilton et al. [7]. We calculated the CN of a random selected animal, dedicated as calibrator, and the CN of all samples were calculated relative to the calibrator. The TSPY CN determined by relative qPCR showed surprisingly low CN, that varied between 2 and 5 copies at the individual, breed and exon levels. It is likely that the main source of this variability is the dependency of calculations on PCR efficiency values. This has been clearly shown when minimally different efficiency values determined by two methods for efficiency calculation (STD curve vs. LinReg PCR) resulted in significantly different copy numbers (Fig 2). Observations by others also pointed the lower precision of qPCR at low copy number range [33,34] and the impact of DNA quality variations among the samples could also not be ruled out.

To further investigate TSPY CN and its CNV status we applied ddPCR, a recently developed technique for absolute quantitation of nucleic acids, to the same samples as used for qPCR [27]. In contrast to qPCR, where the quantitative information is calculated from the parameters of the real-time registered amplification curve (threshold cycle or Ct value and efficiency), ddPCR measures the absolute quantity of DNA in thousands of nanolitre volume reaction partitions (droplets) generated from the homogenous reaction mix. This method employs end-point detection (positive or negative PCR amplifications in each droplet), thus independent of reaction efficiency, which represents a major advantage in our TSPY CN discovery study [33,35]. The ddPCR experiments clearly identified the porcine TSPY as a multicopy gene with three genomic copies (CN = 3). We did not observe any copy number variations among the 20 individual samples or the four breeds investigated. Moreover, no difference was found among the three exons (Fig 3). Although qPCR is the standard and proven technique for relative quantitation, its applicability for DNA CN discovery, especially in the low copy number range, is limited by the lack of a standard sample with known copy number [32].

The low copy non-CNV status makes the pig TSPY similar to the chimpanzee and rhesus monkey, which also have low copy numbers (CN = 6, although CNV status has not been reported), and very different from humans and cattle, both of which have high TSPY copy numbers and show inter-individual variation [6,7]. This copy number variation has also been associated with various reproductive phenotypes. Low TSPY CN was found in men with impaired sperm production [9,36,37], although other human studies with smaller or more heterogeneous donor population reached contradictory results [38,39]. Also, positive correlation was found between TSPY CN and field fertility (adjusted non-return rates) in Holstein and multiple seminal quality parameters of crossbred (Bos taurus × Bos indicus) bulls [19,20]. To further investigate the speculation that the porcine TSPY is not a CNV, we have included five Low-fertility boars with negative DBE scores and five boars with exceptional High-fertility records. Interestingly, there was no difference between the High- and Low-fertile groups, but we detected slightly lower TSPY CN at all three exons (2.56–2.85) in both groups, as compared to normal animals (Fig 3) Most probably this difference would still be interpreted as CN = 3 in most clinical practice [32], which we attributed to technical variability of the tests resulting from the age and quality of these DNA samples that had been banked for several years. Additionally, we could not exclude the biological possibility that the fractional and in some cases statistically different (exons 1, 5) copy numbers are due to somatic mosaicism. Furthermore, although our PCR analyses were designed to amplify all TSPY sequences in the currently unfinished porcine Y-chromosome, the presence of additional as yet to be discovered sequences can not be ruled out.

Evolutionary investigation in humans has suggested that the highly amplified TSPY copy number has been affected by positive selection and might result in selection advantage through some functions in male reproduction [6] the non-variable, tricopy TSPY in boars that we report here for the first time might represent the minimal number of functional copies to maintain fertility.

## Supporting Information

S1 Table Primer and probe sequences, annealing temperature, product sizes and primer efficiencies. Efficiency values determined from either STD curve or as the average of individual LinRegPCR efficiencies.
(XLSX)

S2 Table TSPY CN at three exons by qPCR in 20 boars. At each exon calculations were made using two different primer efficiencies, as determined from either STD curve or as the average of individual LinRegPCR efficiencies
(XLSX)

S3 Table TSPY ddPCR CN ratios at three exons in 20 normal, 5 Low- and 5 High-fertility boars. Direct Boar Effect (DBE) values are included where available.
(XLSX)
